# 3D Path Planning for the Ground Robot with Improved Ant Colony Optimization †

**DOI:** 10.3390/s19040815

**Published:** 2019-02-16

**Authors:** Lanfei Wang, Jiangming Kan, Jun Guo, Chao Wang

**Affiliations:** 1 School of Information and Communication Engineering, Beijing University of Posts and Telecommunications, Beijing 100876, China; wanglanfei@bupt.edu.cn; 2 School of Technology, Beijing Forestry University, Beijing 100083, China; bjfu_wangchao@163.com

**Keywords:** ground robot, path planning, ant colony optimization, 3D space

## Abstract

Path planning is a fundamental issue in the aspect of robot navigation. As robots work in 3D environments, it is meaningful to study 3D path planning. To solve general problems of easily falling into local optimum and long search times in 3D path planning based on the ant colony algorithm, we proposed an improved the pheromone update and a heuristic function by introducing a safety value. We also designed two methods to calculate safety values. Concerning the path search, we designed a search mode combining the plane and visual fields and limited the search range of the robot. With regard to the deadlock problem, we adopted a 3D deadlock-free mechanism to enable ants to get out of the predicaments. With respect to simulations, we used a number of 3D terrains to carry out simulations and set different starting and end points in each terrain under the same external settings. According to the results of the improved ant colony algorithm and the basic ant colony algorithm, paths planned by the improved ant colony algorithm can effectively avoid obstacles, and their trajectories are smoother than that of the basic ant colony algorithm. The shortest path length is reduced by 8.164%, on average, compared with the results of the basic ant colony algorithm. We also compared the results of two methods for calculating safety values under the same terrain and external settings. Results show that by calculating the safety value in the environmental modeling stage in advance, and invoking the safety value directly in the path planning stage, the average running time is reduced by 91.56%, compared with calculating the safety value while path planning.

## 1. Introduction

The robot is one of the greatest inventions of human beings in the twentieth century and has wide applications [1,2,3]. Path planning is a guarantee for robots to carry out various tasks safely and reliably. Nowadays, research hotspots of robot path planning include the shortest path [3,4], being free of obstacle collisions [5,6,7,8], performing in real-time [8,9,10], etc.

The research on 2D path planning technology has already achieved many important results since the last century. Some good two-dimensional path planning modeling methods are still in use now, such as the grid method [11], topological method [12], visibility graph method [13], and so on. There are some typical path planning algorithms, such as the Dijkstra algorithm [14], Prim algorithm [15], simulated annealing algorithm [16], A* algorithm [17], artificial potential field method [18], and the fuzzy logic-control algorithm [19], which are widely used in engineering practice. 

Compared with the planar environment, it is more promising to study the path planning of underwater [20], air [21], and ground environments.

Robot 3D path planning can be divided into two categories, the traditional path planning method and the intelligent path planning method. Traditional path planning includes methods based on virtual potential field or heuristic rules and methods based on mathematical optimization. The former methods are widely used in 2D path planning, especially the harmonic potential field method [21,22] and artificial potential field method [18,23], which have been successfully extended to 3D space. Methods based on mathematical optimization mainly contain the nonlinear classification method of the support vector machine [24], linear programming method, mixed integer linear programming method [25], etc.

The intelligent path planning method has made great progress in robot path planning. There are many applications: genetic algorithms [8,10,26,27], neural networks [27,28], particle swarm optimization [10,29], ant colony optimization, teaching-learning-based optimization [30], etc. Among these, ant colony optimization is based on the research of ants searching for food, which was proposed in the 1990s [31]. It is a heuristic search algorithm that has a successful application in solving the problems of path planning [3,20,32,33,34,35,36,37,38,39] and function optimization [40,41]. Ant colony optimization does well in positive feedback, parallelism, and robustness. At the same time, the ant colony algorithm also has disadvantages of easy falling into a local optimum and being difficult to get away from deadlock. Due to premature convergence of the ant colony algorithm, Stutzle et al. [33] proposed a maximum and minimum ant system algorithm, which can effectively improve the performance of the ant colony algorithm, but its running time is still too long and the results are locally optimal. Due to the information being distributed in different directions, the pheromone released by ants will mislead the decision of other ants, and Lee et al. [34] used a genetic algorithm to optimize ant colony algorithm parameters to realize dynamic path planning. Wang et al. [42] adopted the results of 30 iterations of the ant colony algorithm as initial values and used a simulated annealing genetic algorithm to optimize the paths, which achieves better planning results in similar running time, compared with the results of 250 iterations of the ant colony algorithm. Yang et al. [3] presented a double-layer ant colony algorithm, which combines two algorithms: a parallel elite ant colony optimization and a turning point optimization algorithm. They used these two algorithms sequentially to obtain the optimal path.

As far as the deadlock problem is concerned, some researchers have put forward their approaches. Gao et al. [35] improved the pheromone initialization and state transition probability, but their method can only reduce the possibility of ants falling into a deadlock, which cannot completely avoid the occurrence of it. Mao et al. [36] put forward the corresponding dead angle table. When the ant was trapped in a deadlock, the penalty function can be used to update the trajectory. This method has been proved to be feasible, but it makes the algorithm more complex and has a large space-time overhead. Ravankar et al. [43] proposed a knowledge-sharing system, which could solve the deadlock problem caused by obstacles, making the planned path more effective.

This paper studies the application of ant colony algorithm in robot path planning. The path planning type is global path planning. The 3D terrain for robot work is known. The shapes and positions of obstacles are fixed. Our study object is ground robot path planning. The robot’s ability to climb and cross ditches is weak. The goal is to find the shortest path from the starting point to the end point, considering obstacle avoidance and deadlock prevention. The contributions can be summarized as follows:

- The environment of path planning is three-dimensional space. This paper adopts a 3D grid method for 3D environment modeling. Before using the 3D grid method, a two-step process is implemented, which simplifies the complex 3D terrain.
- This paper designs the basic pheromone update, which uses the combination of a local pheromone update and a global pheromone update. More importantly, this paper improved the method of global pheromone updating, forming an improved pheromone update.
- In this paper, the calculation of safety value function is presented and incorporated into the heuristic function of the ant colony algorithm. This paper also designs two ways to calculate safety values. The first way is to calculate the safety value in the path planning stage. The second way is to calculate the safety value in the environment modeling phase. Finally, it is concluded that calculating the safety value in advance in the environmental modeling stage and calling it directly in the path planning stage (the second way) can reduce the running time of the algorithm and improve the running efficiency.
- This paper adopts a 3D deadlock-free mechanism. When an ant has no next point to choose from, set the pheromone of the current point to 0, and then return to the previous point to find a feasible path. If it is still deadlocked, continue to set to 0 and return. The deadlock problem of robots can be effectively solved by using this mechanism.
- This paper designs two ant colony optimization algorithms: a basic ant colony algorithm and an improved ant colony algorithm. The basic ant colony algorithm adopts a basic pheromone update, and the improved ant colony algorithm adopts an improved pheromone update. The simulation results show that the improved ant colony algorithm outperforms the basic ant colony algorithm in path planning.

The paper is organized as follows. Section 2 researched the method of environmental modeling and adopted a 3D grid method for 3D environment modeling. Section 3 briefly introduced the theory of the ant colony algorithm. In Section 4, we designed a basic pheromone update, which is a mixture of a local pheromone update and a global pheromone update. We proposed an improved pheromone update in order to solve the problem of easily falling into a local optimum. We presented the heuristic function with a safety value introduced and designed two ways of introducing safety values. We adopted a search pattern that combines planes with visual fields and limited the search range of the robot. We adopted a 3D deadlock-free mechanism to avoid the deadlock phenomenon. At the end of Section 4, we designed two kinds of ant colony optimization: a basic ant colony algorithm and an improved ant colony algorithm, based on Section 2, Section 3 and Section 4. Section 5 described the simulation results and analyses. Section 6 concludes the paper.

## 2. 3D Environment Modeling

Environmental modeling is the first step in path planning. The 2D grid method is commonly used for planar environment modeling in 2D path planning. When it is extended to 3D path planning, we could adopt a 3D grid method, which divides the three-dimensional space into several horizontal planes perpendicular to the *Z*-axis and then divides each horizontal plane into grids [37,38].

**Figure 1:**
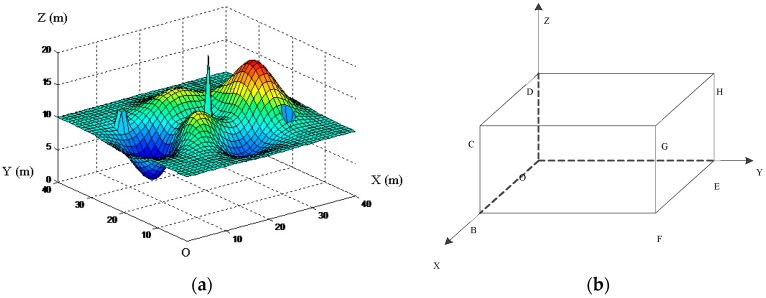
The robot workspace processing. (**a**) The robot workspace after the two-step process; and (**b**) the establishment of 3D planning space.

For the purpose of facilitating the follow-up work and improving the efficiency of path planning, we need to do a two-step process for the robot workspace before using the 3D grid method for modeling. First of all, based on the obstacle-surmounting ability of the robot, we shall remove the obstacles which are lower than the robot striding height, and fill up the gaps which are narrower than the robot striding width. Secondly, to those obstacles and gaps which the robot cannot stride over, we shall do some corresponding expanding treatment according to the robot safe moving radius. An example terrain after the above two-step processing is shown in Figure 1a. This processed terrain is drawn by MATLAB R2014a software, which was produced by the MathWorks, Inc. (Natick, MA, USA). The relevant configurations are described in Section 5.2. In real life, any complex terrain can be processed by the above two-step before the implementation of 3D grid environmental modeling.

Then we shall use the 3D grid method to model the environment. We need to establish a three-dimensional rectangular coordinate system O-XYZ. We construct a 3D region OBCD-EFGH in the coordinate system, in which the plane OBCD is on the plane XOZ, as shown in Figure 1b. We put the terrain after the two-step process (like Figure 1a) in this 3D region, in which the OB side and OE side just equal to the length and width of the robot workspace, respectively.

**Figure 2:**
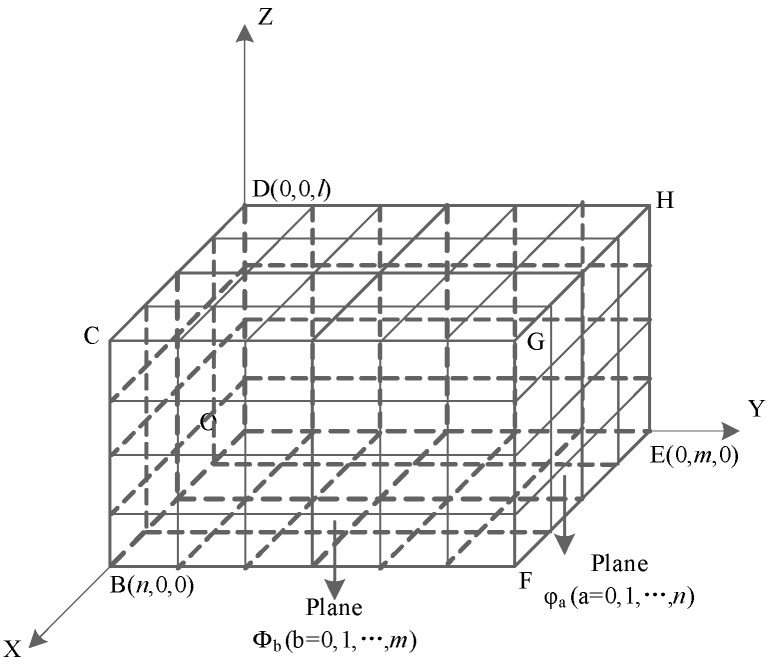
The division of the planning space. The name the plane perpendicular to the *Y*-axis in “Plane $\Phi_{b}(b=0,1,\ldots ,m)$”, and the name of the plane perpendicular to the *X*-axis in “Plane $\varphi_{a}(a=0,1,\ldots ,n)$”.

After obtaining the planning space, we will conduct further division. Firstly, we divide OBCD-EFGH to *m* equal parts along *Y*-axis and create planes parallel to OBCD over every equal-part point, by which we obtain *m* + 1 planes, $\Phi_{b}(b=0,1,2\ldots ,m)$. Then we divide plane $\Phi_{b}(b=0,1,2\ldots ,m)$ to *n* parts along the *X*-axis and *l* parts along the *Z*-axis, by which the planning space will be dispersed to n×m×l cubes, as shown in Figure 2. In practical application, the selection of *m* and *n* should be in terms of the free movement of the robot in a unit grid. As a division of the height, *l* should be selected in terms of the climbing ability of robot. Confirming the values of *m*, *n*, and *l* should also give consideration to the storage of environmental information and the accuracy of path planning. 

In this paper, each terrain has the same size (40 m × 40 m × 20 m). We assume that the length and width of the robot are less than 1 m, the unit grid will be 1 m per side on the plane XOY, and 0.5 m as the unit height along the *Z*-axis. Thus, the grid division is 40 × 40 × 40 in the direction of *X*-axis, *Y*-axis, and *Z*-axis. We set the height of the robot’s obstacle crossing to one unit of grid length (0.5 m), the coordinate difference on the *Z*-axis between the robot’s current position and its next position is no more than one unit length (|Δz|≤0.5 m), which means that robots do not have the ability to raise or drop more than 0.5 m in one step. 

For various sizes of different robots and working environments, the unit grid length in the XOY plane and the *Z*-axis can be reset based on the actual size of the robot, obstacle surmounting ability of the robot, dimensions of obstacles, the height of the slope, the depth of the groove, as well as the size of the robot activity space.

After the treatments above, a set of discrete points represents the robot workspace, which is denoted as $S^{*}$. An arbitrary point A in the set $S^{*}$ can be represented by three-dimensional coordinate A(i,j,k), in which (i={1,2,…,n},j={1,2,…,m},k={1,2,…,l}), and (i,j,k) indicates the location of discrete point A in space. It demonstrates that the distance between two arbitrary points in the set $S^{*}$ can be expressed as:

(1) $$d(A_{1},A_{2})=\sqrt{{\left(x_{1}-x_{2}\right)}^{2}+{\left(y_{1}-y_{2}\right)}^{2}+{\left(z_{1}-z_{2}\right)}^{2}}.$$

The calculation of the robot’s path length can be transformed into the calculation of the sum of the discrete points’ distance, we present the formula as:

(2) $$L=\sum_{a=1}^{q-1}\sqrt{{\left(x_{a+1}-x_{a}\right)}^{2}+{\left(y_{a+1}-y_{a}\right)}^{2}+{\left(z_{a+1}-z_{a}\right)}^{2}},$$

where *q* is a total number of discrete points traveled by the robot.

## 3. An Overview of the Ant Colony Algorithm

### 3.1. The Mathematical Model of Ant Colony Optimization

The mathematical model can be described as found in [44]: Assume the number of ants is *r*, the total number of nodes in the movement of ants is *s*, the distance $d_{ef}$ between node *e* and node *f* (*e*, *f* = 1, 2, …, *s*) is known. The pheromone concentration of the path connected by node *e* and node *f* at *t* time is $\tau_{ef}(t)$. At first, the pheromone concentration of each node connection path is the same ($\tau_{ef}(0)=\tau_{0}$). Ant *g* (*g* = 1, 2, …, *r*) determines its next access node mainly according to the value of pheromone concentration of each node connection path. $p_{ef}^{g}(t)$ represents the probability that ant *g* transfer from node *e* to node *f*, and its calculation is as follows:

(3) $$p_{ef}^{g}=\left\{ \begin{array}{ll} \frac{{\left[\tau_{ef}(t)\right]}^{\alpha }\times {\left[\eta_{ef}(t)\right]}^{\beta }}{\sum_{R\in allow_{g}}\left({\left[\tau_{ef}(t)\right]}^{\alpha }\times {\left[\eta_{ef}(t)\right]}^{\beta }\right)}, & R\in allow_{g}\\ 0, & R\notin allow_{g} \end{array}\right.,$$

where *R* represents all candidate points of the next step of the ant. $\eta_{ef}(t)$ is a heuristic function, $\eta_{ef}(t)=1/d_{ef}$ which expresses the level of expectation that the ants transfer from node *e* to node *f*. $allow_{g}$ (*g* = 1, 2, …, *r*) is a set of nodes that ant *g* can visit. At first, there are (*s* − 1) elements in $allow_{g}$, including all other nodes (except the start node of ant *g*). As time goes on, the elements of the $allow_{g}$ continuous reduction until the end, which means that all the nodes have been visited; α is the pheromone importance factor. β is the heuristic function importance factor. The larger the value of β, the greater the role the heuristic function will be in the transfer. 

### 3.2. Pheromone Update

The pheromone is an information carrier as an attraction to the ants in the process of foraging. It is very important to the algorithm convergence speed and the effect of path planning. After 3D environment modeling, the environment is replaced by a set of discrete points. In order to reduce space expense, we use the discrete points as the carrier of the pheromone. Each discrete point has a corresponding value of pheromone, with higher values having a greater degree of attraction to ants. Accordingly, the local pheromone update and global pheromone update of the ant colony algorithm can be described as follows:

#### 3.2.1. Local Pheromone Update

When an ant passes a discrete point A(i,j,k), it will immediately invoke the corresponding rule to update the pheromone of that discrete point, reducing the pheromone concentration of the point that an ant just passed by. Its purpose is to reduce the possibility of other ants selecting visited points and increase the probability of selecting unvisited points, finally achieving the goal of the search. A local pheromone update is called when an ant passes a point, as:

(4) $$\tau_{ijk}(t+1)=(1-\xi )\tau_{ijk}(t),$$

where $\tau_{ijk}(t)$ indicates the concentration value of the pheromone on the discrete point A(i,j,k), and *t* represents the number of pheromone concentration update, while ξ(0<ξ<1) is pheromone attenuation coefficient.

#### 3.2.2. Global Pheromone Update

After a cycle calculation is completed, use each ant searched path to form an evaluation set, and select the optimal path from the evaluation set, then update the pheromone of the discrete points on the optimal path. Path length calculation method is shown in Equation (2), and the global pheromone update rules are as follows:

(5) $$\tau_{ijk}(t+1)=(1-\rho )\tau_{ijk}(t)+\rho \Delta \tau_{ijk},$$

(6) $$\Delta \tau_{ijk(t)}=\frac{K}{\min(\left\{length(g)\right\})}.$$

Equation (5) represents the pheromone concentration of discrete points in the 3D environment, and ρ(0<ρ<1) indicates the pheromone update coefficient. In Equation (6), {length(g)} indicates a path length set searched by ant *g* (*g* = 1, 2, …, *r*), and *K* is a constant.

## 4. 3D Path Planning with Ant Colony Optimization

We designed two ant colony algorithms in this paper, a basic ant colony algorithm and an improved ant colony algorithm, respectively. The basic ant colony algorithm and the improved ant colony algorithm adopt the same modeling method, which we described in Section 2. The two ant colony algorithms also use the same heuristic function, search pattern, and 3D deadlock-free mechanism. These are all our own designs, which will be explained in Section 4.2.2, Section 4.3, and Section 4.4.2.

The key difference between the basic ant colony algorithm and the improved ant colony algorithm is the pheromone update, which will be first explained in Section 4.1.

### 4.1. Basic and Improved Pheromone Update

The basic ant colony algorithm adopted a basic pheromone update, and improved ant colony algorithm adopted an improved pheromone update. According to the idea of the ant colony algorithm, the two pheromone update methods are designed as shown below.

#### 4.1.1. Basic Pheromone Update

In the basic pheromone update (the basic ant colony algorithm adopted), we used both a local pheromone update and global pheromone update. Local pheromone update requires that every ant must carry out a local pheromone update when it passes by a point in order to reduce the pheromone concentration of that point and to avoid other ants’ repeated selection of that point, so we updated it with Equation (4). However, global pheromone update updates pheromones on the optimal path after all ants have finished traversing, so we updated points on the optimal path with Equation (5) and Equation (6). It is a combination of the local pheromone update and global pheromone update.

#### 4.1.2. Improved Pheromone Update

Consistent with basic pheromone update, our improved pheromone update (improved ant colony algorithm adopted) adopts the combination of local pheromone and global pheromone. As for the local pheromone update, the updating method is the same as the basic ant colony algorithm. See Equation (4).

As the global pheromone update method implements after a complete iteration, and only updates the discrete points on the optimal path. The basic global pheromone update leads to a great increase of the pheromone concentration on the optimal path. As a result, the search will focus on that optimal path, making ants choose that path repeatedly, and the algorithm will turn into a locally optimal solution, limiting the search of the real optimal path. 

Therefore, we made the following improvements in updating global pheromones. We first proposed a new global pheromone update strategy, which not only updates the pheromone concentration of the discrete points on the searched optimal path, but also updates the pheromone concentration of the discrete points on the current optimal path. This strategy can increase the pheromone concentration of points on the actual optimal path and reduce the possibility of falling into a locally optimal solution. 

Equation (6) is the calculation method of pheromone update increment adopted by the basic ant colony algorithm. It leads to a continuous increase in the increments of pheromones. At the beginning of algorithm operation, the pheromone increment value is small, and the pheromone concentration of the discrete points on the optimal path does not increase obviously. In the late stage of algorithm operation, the optimal path length is relatively short, and the pheromone increment is large, so the pheromone concentration of the discrete points on the optimal path increases too much. Meanwhile, the pheromone concentrations of the other discrete points are lower. This will result in a local optimum. Hence, a new method for the calculation of pheromone increment in the global pheromone update process is as follows:

(7) $$\Delta \tau_{ijk}=\frac{\lambda \times (N-M)+K}{\min(\left\{length(g)\right\})},$$

where *N* indicates the maximum iteration number, and *M* indicates the current iteration number of the algorithm. *K* and λ are constants. Through the improvements of pheromone update, the increment of the pheromone is decreasing with the increase of the iteration number of the algorithm. Accordingly, the pheromone concentration on the optimal path increases smaller, which reduces the possibility of other ants choosing the optimal path and increases the possibility of searching for the global optimal solution. When more ants search for the optimal path, this will prevent ants from falling into a locally optimal path. 

### 4.2. Design of a Heuristic Function with Safety Value Introduced

#### 4.2.1. Heuristic Function Design

The heuristic function has an important role in the robot 3D path planning algorithm, just as $\eta_{ef}(t)=1/d_{ef}$ of Equation (3) in Section 2, $\eta_{ef}(t)$ is a heuristic function, aiming at using the heuristic rules to guide the robot from the start point to end point. As our research object is a ground robot with a weak ability to surmount obstacles, the design of the heuristic function should not only consider the shortest path problem but also needs to consider that the ground robot must stick to the ground. Hence, we do not consider points that are more than one grid away from the terrain. According to the above requirements, the designed heuristic function is:

(8) $$\left\{ \begin{array}{l} Q(i,j,k)=U(i,j,k{)}^{w_{1}}\times V(i,j,k{)}^{w_{2}}\times W(i,j,k)\\ U(i,j,k)=\frac{1}{\sqrt{(i-i_{c}{)}^{2}+(j-j_{c}{)}^{2}+(k-k_{c}{)}^{2}}}\\ V(i,j,k)=\frac{1}{\sqrt{(i_{e}-i{)}^{2}+(j_{e}-j{)}^{2}+(k_{e}-k{)}^{2}}}\\ \left(i=\left\{1,2,\ldots ,n\right\},j=\left\{1,2,\ldots ,m\right\},k=\left\{1,2,\ldots ,l\right\}\right) \end{array}\right.,$$

where (i,j,k) indicates the next candidate point for an ant. Q(i,j,k) is the heuristic value of the next candidate point (i,j,k). U(i,j,k) indicates the reciprocal of distance from the current point $\left(i_{c},j_{c},k_{c}\right)$ to the next candidate point (i,j,k), prompting the ant to choose its closer point. V(i,j,k) indicates the reciprocal of distance from the next candidate point (i,j,k) to the destination $\left(i_{e},j_{e},k_{e}\right)$. W(i,j,k) indicates whether the next candidate point (i,j,k) is feasible, where 1 is feasible and 0 is infeasible. $w_{1},w_{2}\in [0,1]$ are coefficients, which represent the importance of U(i,j,k) and V(i,j,k). The method to determine whether the next candidate point is feasible can be determined by Section 4.3.

#### 4.2.2. Introduction of Safety Value Function

Due to the complexity of the 3D environment and the distribution of obstacles, the weak robot should keep away from obstacles and slopes beyond climbing ability. For this purpose, we introduced the safety values of the discrete points, and the safety value is calculated as follows:

(9) $$S(i,j,k)=\frac{v-u}{v},$$

where v indicates the total number of discrete points in the visual area of the point (i,j,k). u indicates the number of infeasible points in the same visual area. Because the problem of feasible points has been considered in the calculation of safety value, S(i,j,k) is used instead of W(i,j,k). Therefore, the designed heuristic function with safety value function introduced is

(10) $$\left\{ \begin{array}{l} \;Q(i,j,k)=U(i,j,k{)}^{w_{1}}\times V(i,j,k{)}^{w_{2}}\times S(i,j,k{)}^{w_{3}}\\ \;(i=\left\{1,2,\ldots ,n\right\},j=\left\{1,2,\ldots ,m\right\},k=\left\{1,2,\ldots ,l\right\}) \end{array}\right..$$

In Equation (10), the calculation of U(i,j,k) and V(i,j,k) are the same as Equation (8). $w_{1},w_{2},\;w_{3}\in [0,1]$ are coefficients which represent the important degree of U(i,j,k), V(i,j,k) and S(i,j,k), respectively.

#### 4.2.3. Two Methods of Introducing a Safety Value

Introducing a safety value calculation in the heuristic function can be easier to avoid obstacles, improving the safety of the robot. However, the increased computation will increase the running time and reduce the efficiency of path planning. Therefore, we designed two methods to introduce safety values to discuss real-time of path planning. The first method is to introduce the calculation of the safety value in the process of path planning, which means the robot calculates the safety value while searching the path. However, algorithm complexity is increased in this way. When ants search for the next point, due to not only needing to scan the visual field to get the next feasible points, but also calculate the safety values of these points. Ant colony algorithm is realized by a large number of iterations of ants, and every ant calculates the safety values of the points in the visual field at every step, which leads to repeated calculations, increased redundancy, and computing time. 

In this regard, the second method is to move the calculation of safety value from the process of path planning. Hence, the second method chooses to calculate the safety values of terrain points and their visual field points in the environmental modeling phase, which can be considered as pre-processing. When ants need to calculate the heuristic value, they just invoke the safety value calculated in the environment modeling phase. In this way, duplication can be avoided.

In Section 5.3, we compare the impact of the two methods of calculating security values on the running time of the algorithm. For a fair comparison, all the running times in this paper are the sum of the time of environmental modeling and the time of path planning.

### 4.3. Search Pattern Design

**Figure 3:**
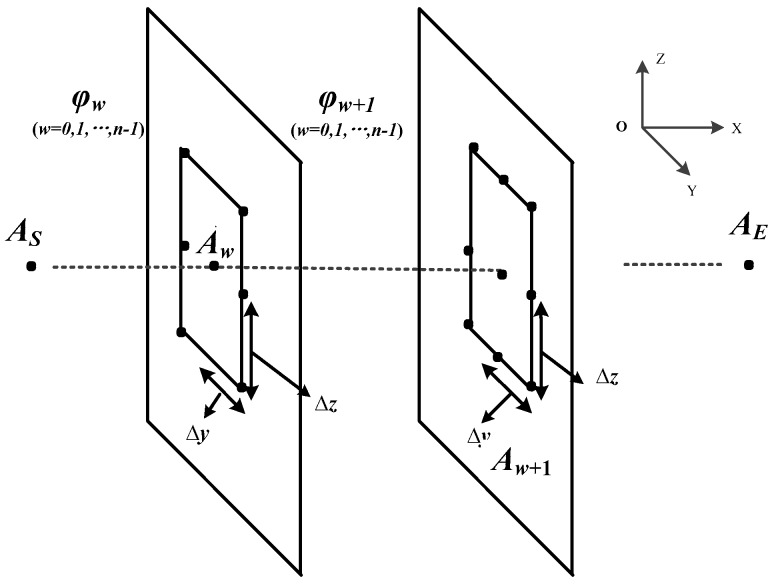
Path selection process. Set the robot’s mobile unit to be 1 grid along the *Y*-axis and *Z*-axis. Δy=±1 m and Δz=±0.5 m.

The search pattern is a mode combining the plane and visual field. We assume that the main direction of robot movement is along the *X* direction. As shown in Figure 3, the robot start point is $A_{S}(i_{S},j_{S},k_{S})$, and the end point is $A_{E}(i_{E},j_{E},k_{E})$, and $\varphi_{w}(w=0,1,2\ldots ,n-1)$ is a plane parallel to YOZ. The robot motion is simplified to three kinds: forward motion, lateral motion, and longitudinal motion. These three motions move along the *X*-axis, *Y*-axis, and *Z*-axis, respectively. When the robot moves a unit grid in the forward motion along *X*-axis, its maximum displacement of lateral motion are allowed to be Δy, and its maximum displacement of longitudinal motion is allowed to be Δz. In this paper, we set Δy=1 m and Δz=0.5 m. For instance, if an ant is at the point $A_{w}(i_{w},j_{w},k_{w})$ on the plane $\varphi_{w}$, there are 17 points in the visual field of point $A_{w}$, and *v* stands for any point in the visual field. Since the study object is a ground robot, six points on the plane $\varphi_{w}$ and nine points on the next plane $\varphi_{w+1}$ are selected as candidate points, represented as large black dots in Figure 3. In these 15 candidate points, the points on the terrain are feasible points. The sum of the number of feasible points and the number of infeasible points is equal to the total number of points in the visual field (17 in this paper).

The steps of an ant at point $A_{w}$ on the current plane $\prod_{w}$ choosing the next point are as follows:

Step1. Determine the visible area of the point $A_{w}$ and identify feasible points.
Step2. Compute the heuristic value {Q(v)} of all the visual points according to the heuristic function, Equation (10).
Step3. Compute the selection probability {P(v)} of all the visual points according to Equation (11):
  (11) $$P(v)=\left\{ \begin{array}{ll} \frac{{\left[\tau_{v}\right]}^{\alpha }\times {\left[Q(v)\right]}^{\beta }}{\sum \left({\left[\tau_{v}\right]}^{\alpha }\times {\left[Q(v)\right]}^{\beta }\right)}, & \mathrm{if}\;v\;\mathrm{is}\;\mathrm{feasible}\;\mathrm{point}\\ 0, & \mathrm{otherwise} \end{array}\right..$$
Step4. Based on every point’s selection probability, use the roulette method to determine the next point $A_{w+1}$.

In Equation (11), $\tau_{v}$ indicates pheromone concentration of the point v. α and β represent the important degree of pheromone concentration and heuristic value, respectively.

### 4.4. Deadlock-Free Mechanism

#### 4.4.1. 2D Deadlock-Free Mechanism 

**Figure 4:**
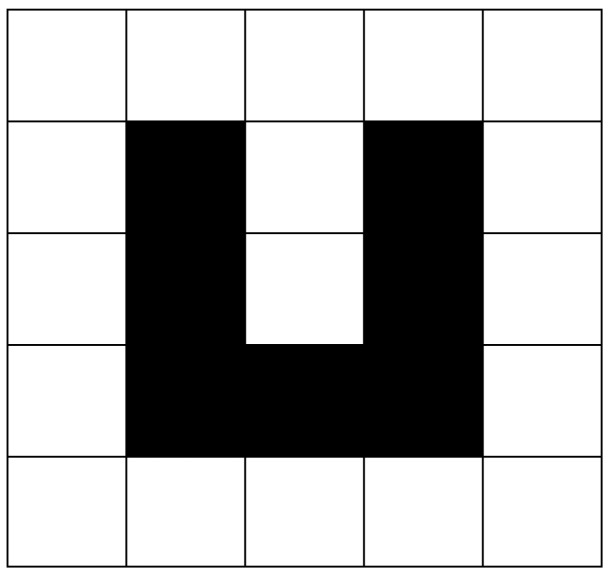
Deadlock phenomenon.

In the process of path planning, robots often fall into a dead end in path searching, surrounded by obstacles, with no choice of a next step, leading robots to lose the possibility of continuing movement. This phenomenon is called “deadlock”, as shown in Figure 4.

**Figure 5:**
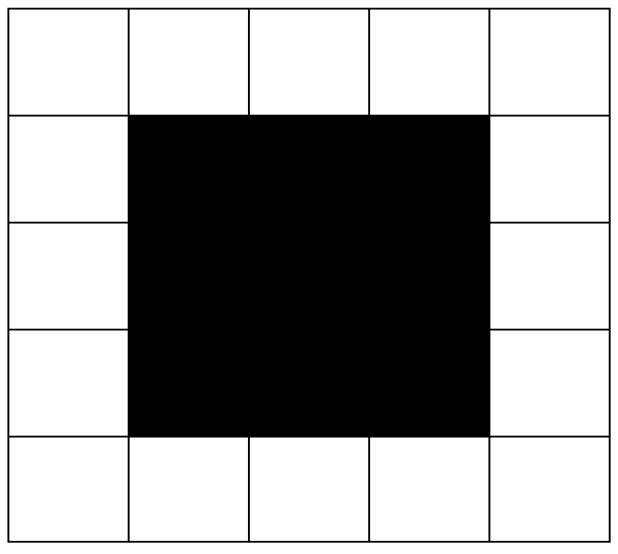
Concave obstacle filling.

In the path planning of a 2D plane, the usual practice is to fill up concave obstacles in the process of environmental modeling [39], as shown in Figure 5. In this way, all obstacles in the 2D plane will be convex obstacles, which can eliminate the trap caused by the concave obstacle and avoid the situation of deadlock.

#### 4.4.2. 3D Deadlock-Free Mechanism 

In the path planning of 3D space, because of the complexity of the land environment and obstacles, and considering the robot’s climbing ability, adding that it is not realistic to be able to locate the traps in the process of environmental modeling, the method of filling the trap is not feasible. Inspired by the idea of information sharing in [43], we adopted a relatively simple method which can effectively avoid the ants falling into deadlocks. When an ant falls into a trap without a feasible point being available, it would set the pheromone of the current point to 0 in order to make the current point infeasible, and return to the previous point. The ant would choose a new point again, if there is still no feasible point, let the ant move one step back, and keep retreating until it escapes the trap. Without knowing the trap information in advance, as long as an ant falls into the trap and pulls itself out, other ants will never fall into that trap. This method is easy to apply, and its efficiency of relieving the deadlock is relatively high.

### 4.5. The Overall Flow of Ant Colony Optimization

As described at the beginning of Section 4, designs of the basic ant colony algorithm and the improved ant colony algorithm are summarized in Table 1.

From Table 1, designs of the basic ant colony algorithm and the improved ant colony algorithm can be clearly seen. Only the global pheromone update strategy is different; the other aspects are the same. Therefore, the steps of the two algorithms can be summarized as follows:

Step1. Model the robot workspace and initialize all parameters. Determine the start point and end point of the robot in 3D space, and put all ants in the start point.
Step2. Set the discrete point ant *g* (*g* = 1, 2, …, *r*) located as center, calculate the probability of selecting every discrete point in the visible field according to Equation (11), and select the next point according to the roulette method.
Step3. Perform local pheromone update for every selected point according to Equation (4).
Step4. Judge whether or not the ant falls into a deadlock. If so, call the 3D deadlock-free mechanism and return to step 2.
Step5. Judge whether or not the whole path of an ant is constructed. If so, plus one to the serial number of the ant.
Step6. Judge whether or not that all ants have completed a path construction. If so, perform global pheromone update, and enter the next loop iteration.
Step7. Judge whether or not the program meets the stagnation conditions. If so, output the results.

**Figure 6:**
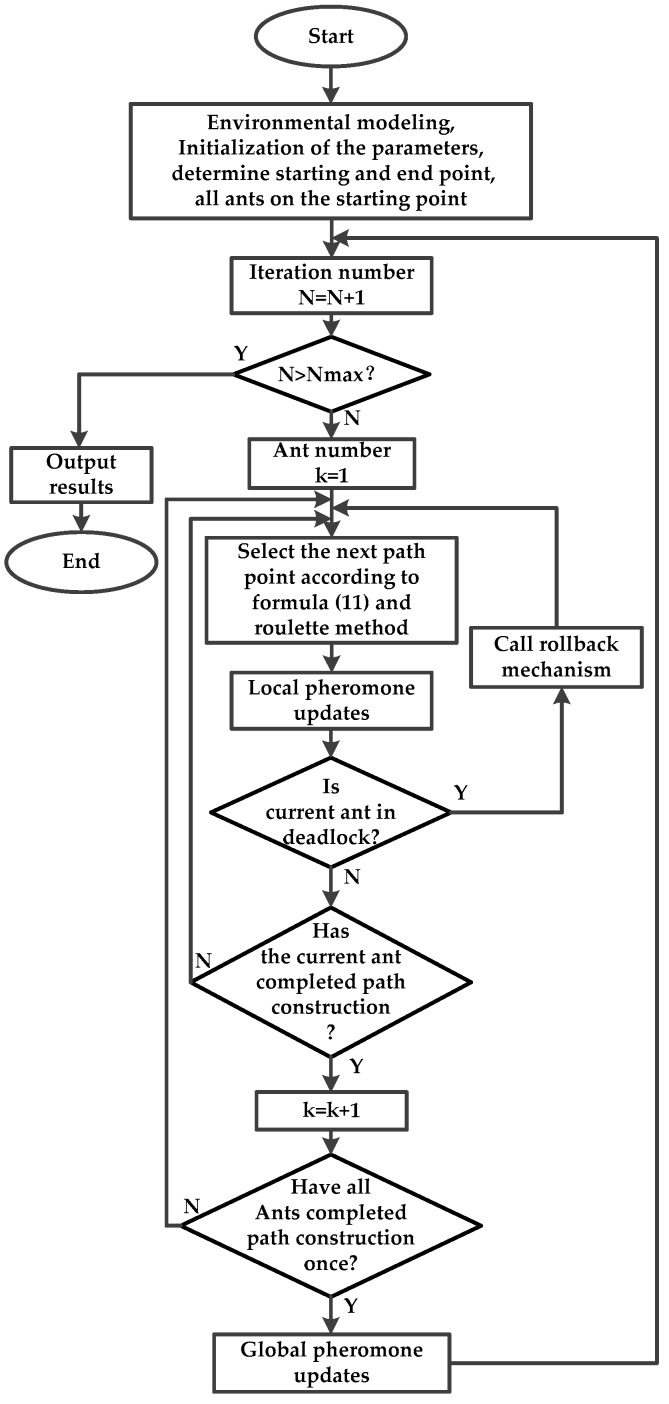
The flow chart of ant colony optimization, which corresponds to the steps above.

The corresponding flow chart of ant colony optimization for ground robot 3D path planning is as shown in Figure 6.

## 5. Simulation Results and Analyses

### 5.1. Analysis and Selection of Algorithm Parameters 

#### 5.1.1. The Choice of Ant Number

In the process of path planning, ants can find a better path through mutual cooperation with each other. Usually, the more ants, the stronger the global search capability, which is better for the stability of the algorithm. However, a larger ant number will decrease the difference of pheromone concentration between paths, leading to the convergence rate of the algorithm slowing down. Conversely, the fewer ants there are, the faster the algorithm convergence will be, but at the same time the global search capability and the stability of the algorithm will be weakened, and the algorithm will be premature. Hence, the ant number should be determined according to the size of a problem [45]. In this paper, considering the robot workspace and the grid density, as well as the cost of the time, and through comparing the simulation operation results of different ant number, we finally set the ant number *r* = 20.

#### 5.1.2. Combination of α, β, ρ and ξ

The important degree factor of pheromone α and β reflected in the operation of the algorithm, representing the important degree of the pheromone accumulated by discrete point and heuristic values of this point in the process of ant transfer. When the α value is greater than β, the empirical factor occupies a major role, and the greater the possibility of ants repeating the walked path, thereby weakening the randomness of the search; when the α value is less than β, the deterministic factor occupies a major role, and ants are eager to choose the path cost minimum synthetically, which accelerates the convergence speed of the algorithm, but makes it easy to fall into a local optimum, ignoring better paths. Similarly, the size of the global pheromone update coefficient ρ and local pheromone attenuation coefficient ξ are directly related to the global searching ability and algorithm convergence speed.

In the actual situation, α, β, ρ and ξ are not independent, but have mutual influence, the comprehensive role [46]. We considered other researchers’ settings and attempted a variety of combinatorial simulations. Finally, we set the values of these four parameters: α=1, β=1, ρ=0.2, and ξ=0.2.

### 5.2. Comparison Simulations of the Basic and Improved Ant Colony Algorithm

For testing the feasibility, effectiveness, and reliability of basic and improved ant colony optimization, we used MATLAB R2014a software to conduct simulations. The computer CPU model is an Intel(R) Core(TM) 2 Quad CPU Q9550, and the internal storage capacity is 4 GB. We took a number of 3D terrains to carry out simulations, and typical figures and the results of the specialized analysis are shown below.

#### 5.2.1. Comparison Simulations about Different Terrains

Firstly, the simulation comparison is carried out in different terrains, with the same starting point and end point. Each terrain has the same size (40 m × 40 m × 20 m), grid division is 40 × 40 × 40, in the direction of *X*-axis, *Y*-axis, and *Z*-axis. We used two ant colony algorithms (basic and improved) for each terrain path planning several times and took the results of two terrains.

**Figure 7:**
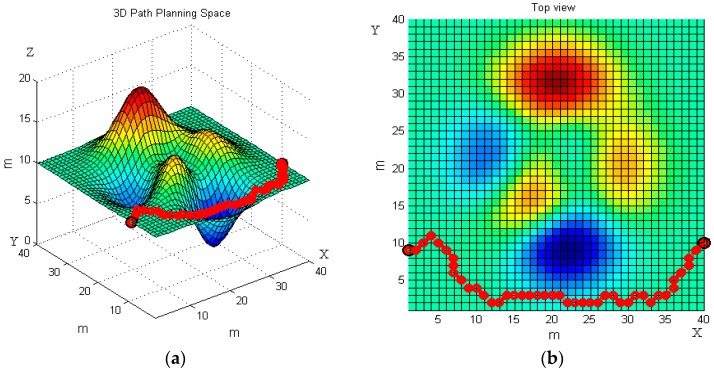
The robot’s moving trajectory using the basic ant colony algorithm in the first terrain. (**a**) Front view; and (**b**) top view.

The 3D map in Figure 7a is the first 3D terrain workspace of the robot. The non-planar parts in the terrain can be understood as obstacles, the obstacles above the horizontal plane are slopes, and the obstacles below the horizontal plane are ditches. Red-colored obstacles are convex, blue-colored obstacles are concave. 

**Figure 8:**
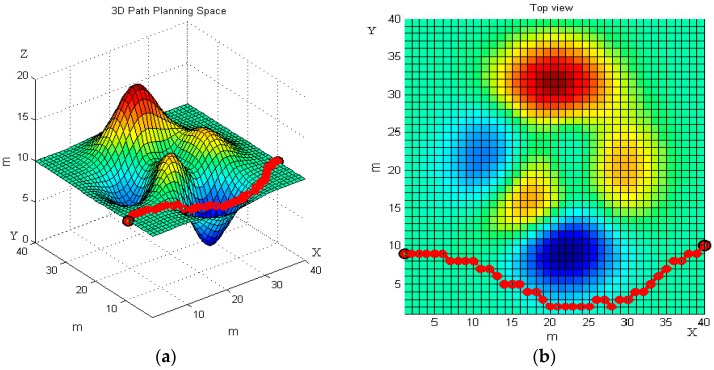
The robot’s moving trajectory using the improved ant colony algorithm in the first terrain. (**a**) Front view; and (**b**) top view.

We set the starting point (1, 9, 20) and the end point (40, 10, 20) on the terrain. The basic ant colony algorithm and the improved ant colony algorithm are used to plan the path of the robot, respectively. Figure 7 and Figure 8 reveal the robot’s moving trajectories. 

Figure 7 and Figure 8 present the average path planning level of the basic and the improved ant colony algorithm. Each figure has two images, the left one is a three-dimensional trajectory, and the right one is its top view. From different views of the robot’s moving trajectories in Figure 7 and Figure 8 we can intuitively see that the improved ant colony algorithm has better performance than the basic ant colony algorithm. The results of the improved ant colony algorithm can effectively avoid obstacles and have no circuitous path. 

**Figure 9:**
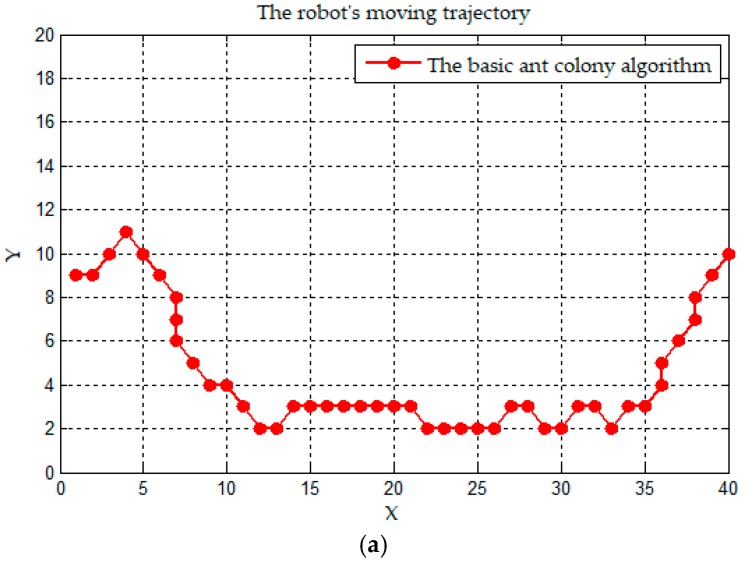

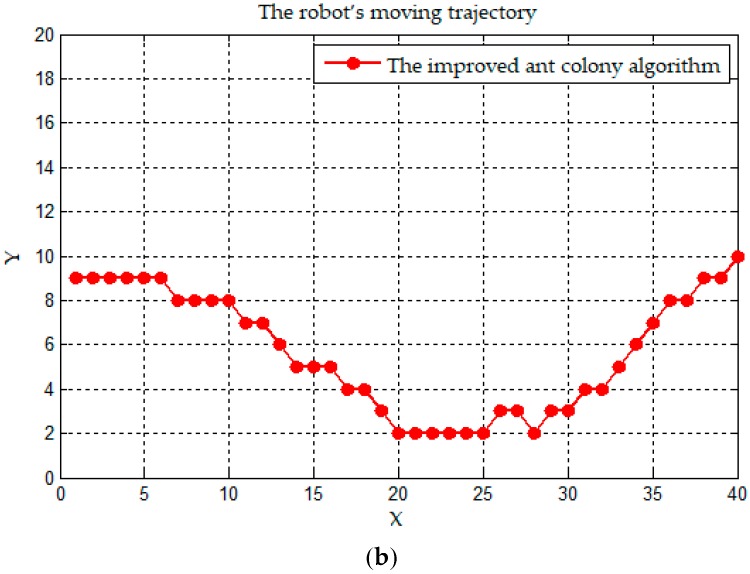
The continuous trajectory mapped on the plane. (**a**) A trajectory of the basic ant colony algorithm; and (**b**) a trajectory of the improved ant colony algorithm.

In order to compare the smoothness of the path trajectories planned by the two algorithms (the basic ant colony algorithm and the improved ant colony algorithm), the path composed of discrete points is processed continuously, inspired by [3,47]. Therefore, both two trajectories can be regarded as continuous, uninterrupted trajectories. The continuous path trajectories are shown in Figure 9.

Figure 9a is the robot’s moving trajectory of the basic ant colony algorithm, and Figure 9b is that of the improved ant colony algorithm. According to the definition of path trajectory in [39], the two trajectories are continuous and uninterrupted, so they satisfy condition $C^{0}$. Both trajectories have inflection points, which we usually call non-derivable points, and neither trajectory satisfies the $C^{1}$ condition. As the number of inflection points in Figure 9a is more than that in Figure 9b, the trajectory of the improved ant colony algorithm is smoother than that of the basic ant colony algorithm.

**Figure 10:**
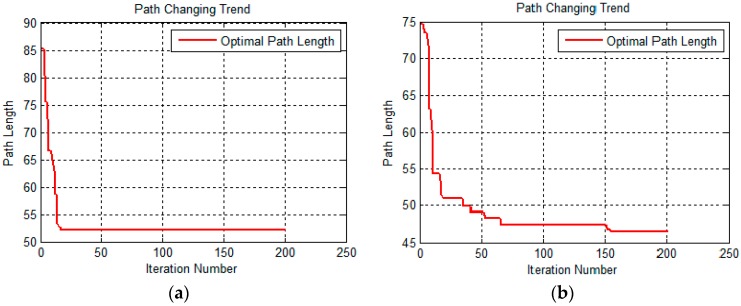
The optimal path length change with iteration number. (**a**) A relation curve of the basic ant colony algorithm; and (**b**) a relation curve of the improved ant colony algorithm.

We also compared the relationship between the optimal path length and the number of iterations. The results are shown in Figure 10.

The two images in Figure 10 represent the optimal path length under different iterations in the form of curves. The image on the left is a relation curve of the basic ant colony algorithm, and the right one is that of the improved ant colony algorithm. The left relation curve drops rapidly to a certain optimum value. After that, the number of iterations increases, the length of the optimal path does not decrease, and the curve shows a horizontal trend. From the data results, we know that the basic colony algorithm gets its optimal path length (52.1648 m, data result) at an iteration number less than 25. However, the relation curve of the improved ant colony algorithm decreases more slowly than that of the basic ant colony algorithm and shows a continuous downward trend as the number of iterations increases. The improved ant colony algorithm gets its optimal path length (47.2646 m, data result) at an iteration number more than 150, and the optimal path is better than the result of the basic ant colony algorithm, which also effectively solves the problem of the basic ant colony algorithm falling into a local optimum. The similar results occur in the second 3D terrain below.

**Figure 11:**
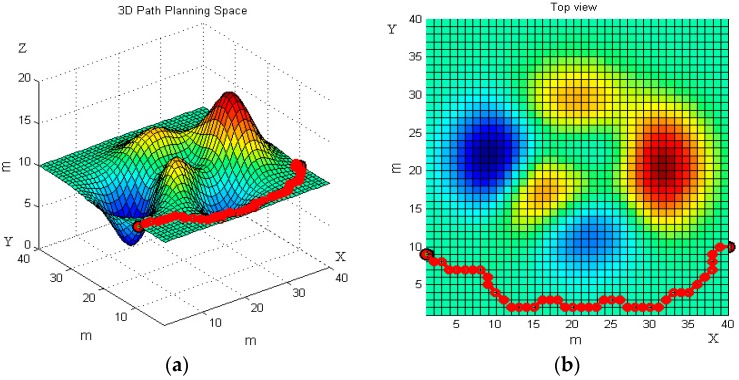
The robot’s moving trajectory using the basic ant colony algorithm in the second terrain. (**a**) Front view; and (**b**) top view.

**Figure 12:**
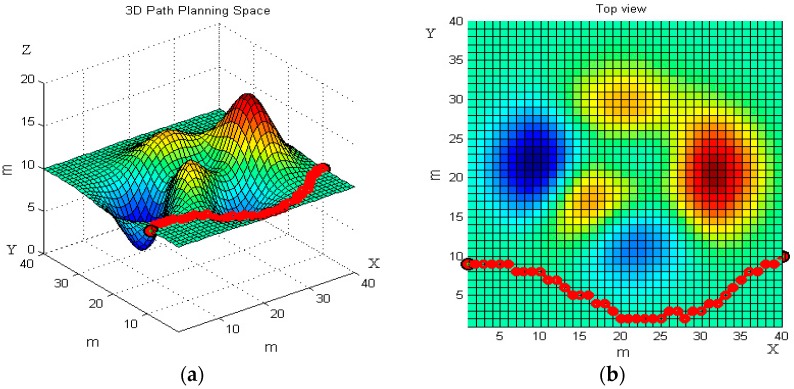
The robot’s moving trajectory using the improved ant colony algorithm in the second terrain. (**a**) Front view; and (**b**) top view.

The 3D map in Figure 11a is the second 3D terrain as the workspace of robot path planning. We set the start point (1, 9, 20) and end point (40, 10, 20), which are the same with the first 3D terrain. The results of the basic ant colony and the improved ant colony on the second 3D terrain are presented in Figure 11 and Figure 12. 

**Figure 13:**
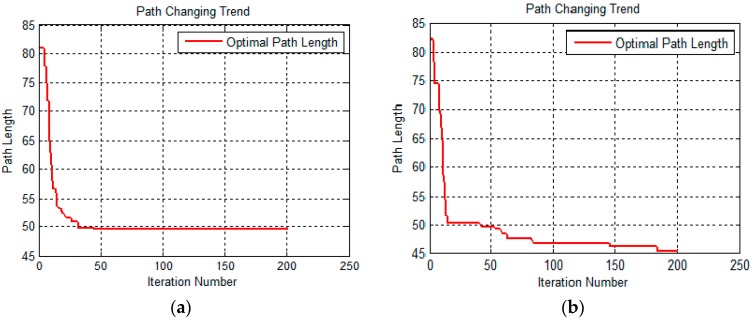
The optimal path length change with iteration number. (**a**) A relation curve of the basic ant colony algorithm; (**b**) A relation curve of the improved ant colony algorithm.

Consistent with Figure 7 and Figure 8, Figure 11 and Figure 12 present the front and top view of the robot’s trajectory. The deeper the red color in the picture, the higher the slope of the obstacle. The darker the blue color, the lower the valley. The same as Figure 10, Figure 13 below is a comparison of the relationship between the optimal path length and the number of iterations. The left one is the result of the basic algorithm, and the right one is that of the improved algorithm.

By comparing the moving trajectory of the robot (Figure 7, Figure 8, Figure 11 and Figure 12), we can see that the results of the improved algorithm, Figure 8 and Figure 12, have much better path planning effects than the results of the basic algorithm, Figure 7 and Figure 11, especially with respect to the shortest path length, avoiding obstacles and trajectory smoothness. From the relationship between the optimal path length and iteration number (Figure 10 and Figure 13), the basic algorithm is prone to premature convergence and local optimum, which cannot obtain the real shortest path.

We selected the running data of the robot path planning of the above two kinds of terrain, and draw two forms which included the shortest path length, running time, and their mean value, as shown in Table 2 and Table 3.

In Table 2 and Table 3, we can see that the average value of the shortest path is shortened from 50.8961 m (the basic ant colony algorithm) to 46.9483 m (the improved ant colony algorithm) in the first 3D terrain, and shortened from 49.9474 m (the basic ant colony algorithm) to 46.3334 m (the improved ant colony algorithm) in the second 3D terrain. The average running time of the basic ant colony algorithm and the improved ant algorithm has very little difference. Results demonstrate that the improved algorithm is obviously more advantageous than the basic algorithm in the aspects of feasibility, effectiveness, and avoiding falling into the local optimum.

#### 5.2.2. Comparison Simulations about Different Starting and End Points

For measuring the reliability of the basic algorithm and the improved algorithm, we chose different starting and end points to conduct simulations. For each starting and end point, we used the basic ant colony algorithm and improved ant colony algorithm to operate multiple times and obtained the average value of the shortest path length and operation time. We took one 3D terrain (the second 3D terrain in the passage above) as an example. Table 4 below is the average operation data of the basic ant colony algorithm and improved ant colony algorithm with different starting and end points. It is worth noting that five situations are cases consisting of five start points and five corresponding end points. From situation 1 to situation 5, the start points are (1, 9, 20), (1, 25, 19), (1, 36, 20), (1, 1, 20), and (1, 38, 20). The corresponding end points are (40, 10, 20), (40, 13, 21), (40, 13, 21), (40, 39, 20), and (40, 2, 20).

From Table 4, we can see that both the basic ant colony algorithm and the improved ant colony algorithm are feasible and their average outputs are stable at a certain level. The average outputs of the improved algorithm are better than that of basic algorithm, and its shortest path length is reduced by 8.164% on average. The average running time differences between the two algorithms are small, and all the differences are within 3 s.

### 5.3. Comparison Simulations of Algorithm Running Time

As for the running (operation) time of the algorithm, the most important factor is the calculation of the safety value in the heuristic function. In Section 4.2.2, we introduced the safety value function into the heuristic function. In Section 4.2.3, we designed two methods to calculate the safety value. The first method is to calculate in the path planning stage, and the second method is to calculate in the environment modeling stage and invoke the calculated safety value directly in the path planning stage. 

In order to verify which of the two methods is more effective, we used the same terrain (the second terrain above), parameters, and settings. We conducted the simulations with the improved ant colony algorithm which implemented the calculation of the safety value in the process of path planning. We also performed the simulations with the improved ant colony algorithm which calculated the safety values of visual points of the points representing the terrain in the stage of the environmental modeling. Through multiple simulations, the typical average running time of the two methods are collected, as shown in Table 5. The running time in Table 5 is the sum of the time of environmental modeling and the time of path planning, and the situation information in Table 5 is the same as Table 4.

From Table 5, we can see that if the safety values of terrain points and their visual field points in planning space have been calculated in the environmental modeling stage, the average running time of the second method can be reduced by 91.56%, compared with the first method. Hence, the improved ant colony algorithm adopts the second method to calculate safety values in the environment modeling stage in advance and calling directly in the path planning stage can greatly reduce the path planning time. This method is feasible, effective, and it has important significance in practical application.

## 6. Conclusions

The main research contents and achievements are as follows:

In this paper, we introduced the path planning research status and discussed the ant colony algorithm application to robot path planning in detail. We adopted a 3D grid method to model the 3D environment. We designed the basic pheromone update which is a combination of a local pheromone update and a global pheromone update. We improved the method of global pheromone update and proposed the improved pheromone update in order to solve the problem of easily falling into a local optimum. We designed a search mode combining planes and visual fields and defined the range of the robot’s movement. At the same time, we redesigned the heuristic function with safety value and adopted a 3D deadlock free mechanism to avoid the deadlock. We also designed a basic ant colony algorithm and an improved ant colony algorithm.

We further analyzed the parameters of the algorithm and designed three kinds of comparative simulations. The relevant conclusions are as follows:

The first two are comparisons between the basic ant colony algorithm and the improved ant colony algorithm, with the same start and end points in different terrains; with different start and end points in the same terrain. We used the basic ant colony algorithm and the improved ant colony algorithm to conduct many simulations and took typical figures and data results for specialized analysis. Through comparing the results of the improved ant algorithm and the basic ant algorithm, we found that the results of the improved ant colony algorithm have much better path planning effects than these of basic ant colony algorithm, especially in avoiding obstacles. The average shortest path length is reduced by 8.164%, compared with the basic ant colony algorithm. The improved ant colony algorithm can effectively solve the most local problems, the planned path is smoother and has fewer inflection points than that of the basic algorithm.

The third comparison is about running time. We used the improved ant colony algorithm to calculate the safety value of the heuristic function in the environmental modeling stage and the path planning stage, respectively. In the same parameter settings, with the same terrain, the results of different starting points and end points show that calculating the safety value in the environmental modeling stage can greatly shorten the running time, and it could raise the efficiency of the algorithm by 12 times. The method of calculating the safety value in advance ensures the 3D path planning in real-time.

## Figures and Tables

**Table 1:** Designs of the basic ant colony algorithm and the improved ant colony algorithm.

Algorithm	Environment Modeling	Pheromone Update (Local and Global)	Heuristic Function	Search Pattern	Deadlock-Free Mechanism
Basic ant colony	3D grid method (Section 2)	Basic pheromone update (Section 4.1.1)	Introducing safety value calculation (Section 4.2.2)	Plane and visual field (Section 4.3)	3D deadlock-free mechanism
Improved ant colony	3D grid method (Section 2)	Improved pheromone update (Section 4.1.2)	Introducing safety value calculation (Section 4.2.2)	Plane and visual field (Section 4.3)	3D deadlock-free mechanism

**Table 2:** The running data of the first terrain.

Method	Indexes Compared	First Time	Second Time	Third Time	Fourth Time	Fifth Time	Six Time	Seven Time	Eight Time	Mean Value
Basic ant colony algorithm	Shortest path (m)	52.164	52.106	50.151	50.042	50.106	49.750	50.685	52.165	50.896
	Running time (s)	110.588	106.861	108.866	83.001	109.967	110.757	111.298	103.301	105.580
Improved ant colony algorithm	Shortest path (m)	47.246	47.704	45.029	46.449	47.990	47.200	46.704	47.265	46.948
	Running time (s)	102.989	103.894	102.637	100.606	106.895	102.951	104.442	111.140	104.444

**Table 3:** The running data of the second terrain.

Method	Indexes Compared	First Time	Second Time	Third Time	Fourth Time	Fifth Time	Six Time	Seven Time	Eight Time	Mean Value
Basic ant colony algorithm	Shortest path (m)	49.547	49.035	47.565	52.165	52.160	48.850	50.151	50.106	49.947
	Running time (s)	104.159	106.549	104.688	107.948	82.357	106.348	106.124	104.700	102.859
Improved ant colony algorithm	Shortest path (m)	46.418	46.449	46.449	46.704	45.232	46.482	46.857	46.080	46.333
	Running Time (s)	103.878	102.094	101.990	104.737	104.391	105.057	102.881	104.836	103.733

**Table 4:** The running data of the second 3D terrain in different situations.

Environment	Method	Indexes Compared	Situation 1 Average Data	Situation 2 Average Data	Situation 3 Average Data	Situation 4 Average Data	Situation 5 Average Data
The second 3D terrain	Basic ant colony algorithm	Shortest path (m)	49.9474	57.3438	61.4708	65.4551	63.6704
		Running time (s)	102.8592	109.9902	116.9173	95.7429	118.8622
	Improved ant colony algorithm	Shortest path (m)	46.3339	51.0887	55.3903	62.5872	58.311
		Running time (s)	103.7331	107.7909	113.3931	93.8504	115.476

**Table 5:** Comparison of algorithm running time.

Introduced Safety Values	Index	Situation 1 Average Data	Situation 2 Average Data	Situation 3 Average Data	Situation 4 Average Data	Situation 5 Average Data
The first method: safety values calculation in the path planning stage	Running time (s)	1232.3486	1221.2706	1313.0922	1150.6062	1411.1165
The second method: safety values calculation in the environment modeling stage	Running time (s)	103.7331	107.7909	113.3931	93.8504	115.4760
